# Genomic evaluations with many more genotypes

**DOI:** 10.1186/1297-9686-43-10

**Published:** 2011-03-02

**Authors:** Paul M VanRaden,, Jeffrey R O'Connell,, George R Wiggans, Kent A Weigel

**Affiliations:** 1 Animal Improvement Programs Laboratory, USDA, Building 5 BARC-West, Beltsville, MD 20705-2350, USA; 2 University of Maryland School of Medicine, Baltimore, MD, 21201, USA; 3 University of Wisconsin, Madison, WI, 53706, USA

## Abstract

**Background:**

Genomic evaluations in Holstein dairy cattle have quickly become more reliable over the last two years in many countries as more animals have been genotyped for 50,000 markers. Evaluations can also include animals genotyped with more or fewer markers using new tools such as the 777,000 or 2,900 marker chips recently introduced for cattle. Gains from more markers can be predicted using simulation, whereas strategies to use fewer markers have been compared using subsets of actual genotypes. The overall cost of selection is reduced by genotyping most animals at less than the highest density and imputing their missing genotypes using haplotypes. Algorithms to combine different densities need to be efficient because numbers of genotyped animals and markers may continue to grow quickly.

**Methods:**

Genotypes for 500,000 markers were simulated for the 33,414 Holsteins that had 50,000 marker genotypes in the North American database. Another 86,465 non-genotyped ancestors were included in the pedigree file, and linkage disequilibrium was generated directly in the base population. Mixed density datasets were created by keeping 50,000 (every tenth) of the markers for most animals. Missing genotypes were imputed using a combination of population haplotyping and pedigree haplotyping. Reliabilities of genomic evaluations using linear and nonlinear methods were compared.

**Results:**

Differing marker sets for a large population were combined with just a few hours of computation. About 95% of paternal alleles were determined correctly, and > 95% of missing genotypes were called correctly. Reliability of breeding values was already high (84.4%) with 50,000 simulated markers. The gain in reliability from increasing the number of markers to 500,000 was only 1.6%, but more than half of that gain resulted from genotyping just 1,406 young bulls at higher density. Linear genomic evaluations had reliabilities 1.5% lower than the nonlinear evaluations with 50,000 markers and 1.6% lower with 500,000 markers.

**Conclusions:**

Methods to impute genotypes and compute genomic evaluations were affordable with many more markers. Reliabilities for individual animals can be modified to reflect success of imputation. Breeders can improve reliability at lower cost by combining marker densities to increase both the numbers of markers and animals included in genomic evaluation. Larger gains are expected from increasing the number of animals than the number of markers.

## Background

Breeders now use thousands of genetic markers to select and improve animals. Previously only phenotypes and pedigrees were used in selection, but performance and parentage information was collected, stored, and evaluated affordably and routinely for many traits and many millions of animals. Genetic markers had limited use during the century after Mendel's principles of genetic inheritance were rediscovered because few major QTL were identified and because marker genotypes were expensive to obtain before 2008. Genomic evaluations implemented in the last two years for dairy cattle have greatly improved reliability of selection, especially for younger animals, by using many markers to trace the inheritance of many QTL with small effects.

More genetic markers can increase both reliability and cost of genomic selection. Genotypes for 50,000 markers now cost <US$200 per animal for cattle, pigs, chickens, and sheep. Lower cost chips containing fewer (2,900) markers and higher cost chips with more (777,000) markers are already available for cattle, and additional genotyping tools will become available for cattle and other species in the near future. All three billion DNA base pairs of several Holstein bulls have been fully sequenced and costs of sequence data are rapidly declining.

Reliabilities of genomic predictions were compared in previous studies for up to 50,000 actual or 1 million simulated markers. Reliabilities for young animals increased gradually as marker numbers increased from a few hundred up to 50,000 [1-3], and increased slightly when markers with low minor allele frequency were included [4]. For low- to medium-density panels (300 to 3,000 markers), selection of markers with large effects preserves more reliability if only the selected markers are used in the evaluation [5], but evenly spaced markers preserve more reliability for all traits if imputation is used [6]. Reliabilities increased from 81 up to 83% as numbers of simulated markers increased from 50,000 to 100,000 using 40,000 predictor bulls [7], however, base population alleles in that study were in equilibrium rather than disequilibrium.

Increasing marker numbers above 20,000 up to 1 million linked markers resulted in almost no gains in reliability in a simulation of 10 chromosomes and 1,500 QTL [8]. Larger gains resulted in a simulation of only one chromosome containing three to 30 QTL that accounted for all of the additive variance [9]. Many genome-wide association studies of human traits have combined large numbers of markers from different chips [10], but those studies almost always estimated effects of individual loci rather than included all the loci to estimate the total genetic effect.

Many genotypes will be missing in the future when data from denser or less dense chips are merged with current genotypes from 50,000-marker chips or when two different 50,000-marker sets are merged, as is being done in the EuroGenomics project [11,12]. Missing genotypes of descendants can be imputed accurately using low-density marker sets if ancestor haplotypes are available [13-15]. At low marker densities, haplotypes provide higher accuracy than genotypes when included in genomic evaluation [1,16]. Missing genotypes were not an immediate problem with data from a 50,000-marker set because >99% of genotypes were read correctly [17].

Fewer markers can be used to trace chromosome segments within a population once identified by high-density haplotyping. Without haplotyping, regressions could simply be computed for available SNP and the rest disregarded. With haplotyping, effects of both observed and unobserved SNP can be included. Transition to higher density chips will require including multiple marker sets in one analysis because breeders will not re-genotype most animals.

Simulated genotypes and haplotypes can be more useful than real data to test programs and hypotheses. Examples are analyses of larger data sets than are currently available or comparison of estimated haplotypes with true haplotypes, which are not observable in real data. Most simulations begin with all alleles in the founding generation in Hardy-Weinberg equilibrium and then introduce linkage disequilibrium (LD) using many non-overlapping generations of hypothetical pedigrees [18] or fewer generations of actual pedigree [19]. Simulations can also include selection [20] or model divergent populations such as breeds [21]. Many genomic evaluation studies simulated shorter genomes and fewer chromosomes than in actual populations, presumably because computing times for obtaining complete data were too long.

Goals of this study are to 1) impute genotypes using a combination of population and pedigree haplotyping, 2) compute genomic evaluations with up to 500,000 simulated markers, and 3) evaluate potential gains in reliability from increasing numbers of markers.

## Methods

### Haplotyping program

Unknown genotypes can be made known (imputed) from observed genotypes at the same or nearby loci of relatives using pedigree haplotyping or from matching allele patterns (regardless of pedigree) using population haplotyping. Haplotypes indicate which alleles are on each chromosome and can distinguish the maternal chromosome provided by the ovum from the paternal chromosome provided by the sperm. Genotypes indicate only how many copies of each allele an individual inherited from its two parents.

Fortran program findhap.f90 was designed to combine population and pedigree haplotyping. Genotypes were coded numerically as 0 if homozygous for the first allele, 2 if homozygous for the second allele, and 1 if heterozygous or not known; haplotypes were coded as 0 for the first allele, 2 for the second allele, and 1 for unknown to simplify matching. The algorithm began by creating a list of haplotypes from the genotypes in the first pass, and the process was iterated so genotypes earlier in the file could be matched again using haplotype refinements that occurred later.

Steps used in the population haplotyping algorithm were: 1) each chromosome was divided into segments of about 500 markers each when analyzing the 500,000 marker or mixed datasets and 100 markers each for 50,000 marker data; 2) the first genotype was entered into the haplotype list as if it was a haplotype; 3) any subsequent genotypes that shared a haplotype were then used to split the previous genotypes into haplotypes; 4) as each genotype was compared to the list, a match was declared if no homozygous loci conflicted with the stored haplotype; 5) any remaining unknown alleles in that haplotype were imputed from homozygous alleles in the genotype; 6) the individual's second haplotype was obtained by subtracting its first haplotype from its genotype, and the second haplotype was checked against remaining haplotypes in the list; 7) if no match was found, the new genotype (or haplotype) was added to the end of the list. Unknown alleles in the genotype were stored as unknown alleles in the haplotype; 8) the list of currently known haplotypes was sorted from most to least frequent as haplotypes were found for efficiency and so that more probable haplotypes were preferred.

**Figure 1:**
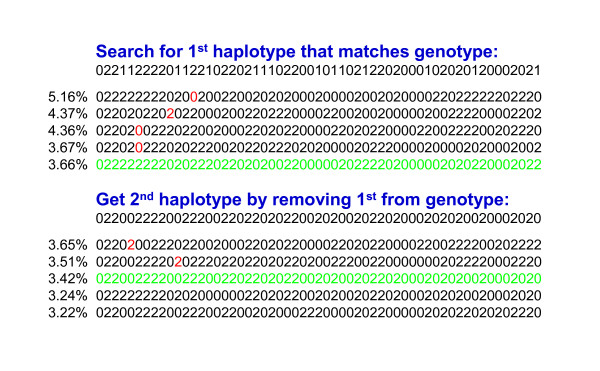
**Demonstration of algorithm to find first and second haplotypes**.

Steps 4) and 6) of the algorithm for population haplotyping are demonstrated in Figure 1 for a shortened segment of 57 markers. The example genotype conflicted with the first four listed haplotypes but had no conflicts with haplotype number 5. After removing haplotype 5 from the genotype to obtain the animal's complementary haplotype, the algorithm searched for the complementary haplotype in the remainder of the list until it was identified as haplotype 8. Instead of storing all 57 codes from the segments found, this animal's haplotypes were stored simply as 5 and 8. In practice, some alleles in the least frequent haplotypes remain unknown because few or no matches were found or because each matching genotype happened to be heterozygous at that locus.

Iteration proceeded as follows. The first two iterations used only population haplotyping and not the pedigree. The first used only the highest density genotypes, and later iterations used all genotypes. The third and fourth iterations used both pedigree and population methods to locate matching haplotypes. Known haplotypes of genotyped parents (or grandparents if parents were not genotyped) were checked first, and if either of the individual's haplotypes were not found with this quick check then checking restarted from the top of the sorted list. For example, the algorithm in Figure 1 could check haplotypes 5 and 8 first if parent genotypes are known to contain these haplotypes. The last two iterations did not search sequentially through the haplotype list and instead used only pedigrees to impute haplotypes of non-genotyped ancestors from their genotyped descendants, locate crossovers that created new haplotypes, and resolve conflicts between parent and progeny haplotypes. If parent and progeny haplotypes differed at just one marker, the difference was assumed to be genotyping error, and the more frequent haplotype was substituted for the less frequent.

Imputation success was measured in several ways. Percentages of alleles missing before and after imputation indicated the amount of fill needed and remaining. Percentages of incorrect genotypes were calculated across all loci including the genotypes observed, the haplotypes imputed, and the remaining haplotypes not imputed but simply assigned alleles using allele frequency. An alternative error rate counted differences between heterozygous and homozygous genotypes as only half errors and differences between opposite homozygotes as full errors across the imputed and assigned loci but not including the observed loci [11]. The percentage of true linkages between consecutive heterozygous markers that differed from estimated linkages was determined, as well as the percentage of heterozygous loci at which the allele estimated to be paternal was actually maternally inherited.

### Simulating linkage disequilibrium

Methods to simulate LD were derived and the simulation program of [19] was modified to generate LD directly in the earliest known ancestors in the pedigree (the founding population). Previously, marker alleles were simulated in equilibrium and uncorrelated across loci in the founding population, but genotypes at adjacent markers become more correlated as marker densities increase. Most other studies [18] used thousands of generations of random mating to establish a balance between recombination, drift, and mutation in small populations with actual size set equal to effective size. Fewer rare and more common haplotypes would occur than in actual populations with unbalanced contributions to the next generation. Neither the standard nor the new approach may provide exactly the same LD pattern as in actual genotypes.

Initial LD was generated by establishing marker properties for the population, simulating underlying, unobservable, linked bi-allelic markers that each have an allele frequency of 0.5, and setting minor allele frequencies for observed markers to <0.5 by randomly replacing a corresponding fraction of the underlying alleles by the major allele.

Direction of linkage phase for each marker with the previous marker was set to positive (coupling) or negative (repulsion) with 0.5 probability, and this process was repeated across each chromosome. Marker alleles were coded as 1 or 2 and their frequencies were distributed uniformly between 0 and 1. After establishing these initial marker properties, each founding haplotype from an unknown founder parent was generated as follows: 1) for the first locus on each chromosome, an underlying allele was chosen randomly with 0.5 frequency; 2) subsequent loci on the same chromosome were set to the same allele or opposite allele based on direction of initial linkage phase until a break point occurred; 3) if a uniform variate exceeded the LD decay parameter defined as 1 - the fraction of recombinations that had occurred between adjacent loci, then that haplotype block ended and the next allele was chosen randomly with 0.5 frequency; and 4) observed alleles were obtained from the underlying alleles using the allele frequencies. A uniform number was generated at the beginning of each block, and underlying alleles within the block were replaced by the major allele if the minor allele frequency was greater than twice the minor allele frequency at that locus.

The benefit of the underlying markers is that a single parameter can model the gradual decay of linkage disequilibrium as marker distances increase, similar to an autoregressive correlation structure. The idea is similar to using underlying normal variables for categorical traits because the math is simpler on the underlying scale. Each allele in the founding haplotypes required generating only two uniform random numbers: one to determine underlying LD blocks and a second to increase frequency of the major allele. The LD blocks mimic segments preserved from unknown generations prior to the pedigree. The simulation process resulted in different lengths, locations of breakpoints, and patterns of rare alleles for each founding haplotype segment.

### Simulated data

The population simulated included 8,974 progeny-tested bulls, 14,061 young bulls, 4,348 cows with records and 6,031 heifers, as well as 86,465 non-genotyped ancestors in the pedigrees. The founding animals were mostly born before 1960, about 10 generations ancestral to the current population. This population structure was identical to the 33,414 Holstein animals with BovineSNP50 genotypes in the North American database as of January 2010. Many of these animals share long haplotypes because, for example, three bulls each had >1,000 genotyped progeny in the dataset.

Genotypes for 500,000 markers were simulated, and the 50,000 marker subset was constructed using every 10th marker. The simulated percentages of missing genotypes and incorrect reads were 1.00 and 0.02%, respectively, based on rates observed for the BovineSNP50 chip. The LD decay parameter for adjacent underlying alleles was set to 0.998, with an average of 16,667 markers per chromosome, spaced randomly. Linkage disequilibria derived from the simulated and from real genotypes were compared by squared correlations of marker genotypes plotted against physical distance between markers. The haplotyping algorithm was tested using a single simulated chromosome with a length of 1 Morgan, which is the average length for cattle chromosomes. Gains in reliability from genomic evaluation were tested using sums of estimated allele effects across all 30 simulated chromosomes.

True haplotypes from the simulation allow proportions of correctly called linkage phases and paternal allele origins to be checked. Correct calls were summarized for each animal to determine how successful the algorithm was for different members of the pedigree. These estimates of genotype or haplotype accuracy from simulation are needed because true values are not available for comparison with real data. Genotypes, linkage phases and haplotypes were estimated for all animals and compared with their true genotypes and haplotypes from simulation. For each heterozygous marker, paternity was considered to be correctly called if the allele presumed to be from the sire was actually from the sire. Linkage phase was considered to be correctly called if estimated phase matched true phase for each adjacent pair of heterozygous markers.

Effects of quantitative trait loci (QTL) were simulated with a heavy-tailed distribution. Standard, normal effects (*s*) were converted to have heavy tails using the function 2^abs(s - 2)^. The locus with the largest effect contributed 2 to 4% of the additive genetic variance across five replicates, and the number of QTL was 10,000, which is greater than the 100 QTL used previously [19]. Small advantages of nonlinear over linear models for dairy cattle traits indicate many more QTL than previously assumed in most simulations. Similarly, human stature is very heritable (i.e. 0.8) but the 50 largest SNP effects account for only 5% of the variance [22]. If a few large QTL do exist, these causative mutations could be selected for directly instead of increasing density of markers everywhere.

Five replicates of the simulated data were analyzed as five traits, and QTL effects for each trait were independent. Just one set of genotypes contained the five QTL replicates for efficiency as in [19]. All QTL were located between the markers; none of the markers had a direct effect on the traits. Error variance for each genotyped animal was calculated from the reliability of its traditional milk yield evaluation, which for cows might include only one or a few records with a 30% heritability but for bulls could include hundreds or thousands of daughter records. Daughter equivalents from parents were removed from total daughter equivalents to obtain reliability from own records and progeny (REL_prog_), and error variance for each animal equalled additive genetic variance times the reciprocal of reliability minus one, i.e. σ_a_^2 ^(1/REL_prog _-1).

Two mixed density data sets were simulated, which included genotypes from both 500,000- and 50,000- marker chips, to determine if a few thousand higher density genotypes would be sufficient to impute, using program findhap.f90, the missing genotypes for the other animals genotyped with 50,000 markers. The first analysis included 1,406 randomly chosen young bulls with 500,000 markers and the other 32,008 animals with 50,000 markers. The second analysis had 3,726 bulls with 500,000 markers, including 2,140 older bulls that had 99% reliability plus the same 1,406 young bulls, and the other 29,788 animals had 50,000 markers.

### Genomic evaluation

The vector of observed, deregressed observations (**y**) was modelled with an overall mean (Xb), genotypes minus twice the base allele frequency (Z) multiplied by allele effects (**u**), a vector of polygenic effects for genotyped animals (**p**), and a vector of errors (**e**) with differing variance depending on REL_prog_:

y=Xb+Zu+p+e

To solve for polygenic effects, equations for all ancestors of the genotyped animals are included along with **p**, so that the simple inverse for pedigree relationships could be constructed [23]. Reliabilities of solutions for Zu + **p **were obtained from squared correlations of estimated and true breeding values and averaged across five replicates for 14,061 young bull predictions.

Dense markers account for most but not all of the additive genetic variation, and the remaining fraction of variance is the polygenic contribution (*poly*) assumed to be 10 and 0% of genetic variance with 50,000 and 500,000 markers, respectively. Values of *poly *have been assumed to equal from 0 to 20% of additive genetic variance in most national evaluations of actual 50,000-marker data; *poly *should increase with fewer or decrease with more available markers. An initial test with 500,000 markers indicated a 0.1% decrease in reliability and slower convergence with 5% *poly *as compared to 0% *poly *in the model.

Linear and nonlinear models were both applied to the simulated data using the same methods as [24]. The nonlinear model was analogous to Bayes A [9], and a range of values was tested for the parameter controlling the shape of the distribution for both marker densities.

### Reliability approximation

Approximate reliability formulas are needed because correlations of true breeding value (BV) with genomic estimated breeding value (GEBV) are not available in actual data. The maximum genomic reliability that can be obtained in practice (REL_max_) is limited by the maximum marker density and by the size of the reference population. As the reference population becomes infinitely large, reliability should approach 1 minus *poly *because *poly *is the residual QTL variance not traceable by the markers on the chip.

Total daughter equivalents (DE_max_) from the reference population can be obtained by summing traditional reliabilities (REL_trad_) minus the reliabilities of parent average (REL_pa_), multiplying by the ratio of error to sire variance (*k*), and dividing by the equivalent reference size (*n*) needed to achieve 50% genomic REL [25]:

$${\text{DE}}_{\max}=\sum \left({\text{REL}}_{\text{trad}}-{\text{REL}}_{\text{pa}}\right)k/n.$$

Genomic reliabilities for individual animals can account for their traditional reliabilities, numbers of markers genotyped, quality of imputation, and relationship to the reference population. Animals that are less or more related to the reference population may have lower or higher DE_max_. Accounting for individual relationships is automatic with inversion [19] or can be approximated without inversion using elements of the genomic relationship matrix [4,26].

Conversion of DE_max _to genomic REL should account for the fact that genotyped SNP do not perfectly track all QTL in the genome if full sequences are not available. Multiplication by 1 - *poly *prevents reliability to reach 100%. If all reference animals are genotyped at the highest chip density, the expected genomic REL for young animals without pedigree information can be calculated as:

$${\text{REL}}_{\text{max}}=\left(\text{1}-poly\right){\text{DE}}_{\text{max}}/\left({\text{DE}}_{\text{max}}+k\right).$$

Each animal's traditional REL is converted to daughter equivalents (DE_trad_), and these are added to DE_max _adjusted for any additional error introduced by genotyping at lower SNP density. The reduced daughter equivalents from genomics (DE_gen_) can be calculated from the squared correlation between estimated and true genotypes averaged across loci (REL_snp_) for each animal as:

$${\text{DE}}_{\text{gen}}=k\;{\text{REL}}_{\text{max}}\;{\text{REL}}_{\text{snp}}/\left(\text{1}-{\text{REL}}_{\text{max}}\;{\text{REL}}_{\text{snp}}\right)$$

The animal's total reliability REL_tot _is computed from the sum of the daughter equivalents as:

$${\text{REL}}_{\text{tot}}=\left({\text{DE}}_{\text{trad}}+{\text{DE}}_{\text{gen}}\right)/\left({\text{DE}}_{\text{trad}}+{\text{DE}}_{\text{gen}}+k\right)$$

## Results

### Genotype simulation

**Figure 2:**
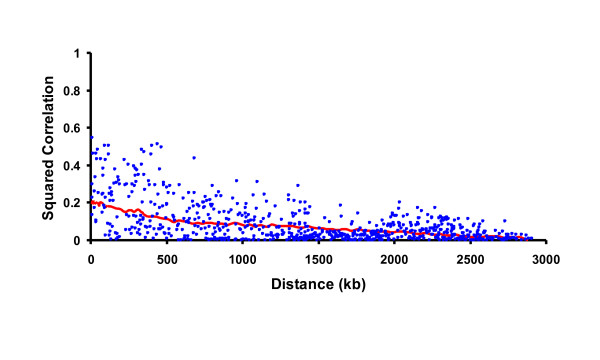
**Linkage pattern among markers on a simulated chromosome**.

**Figure 3:**
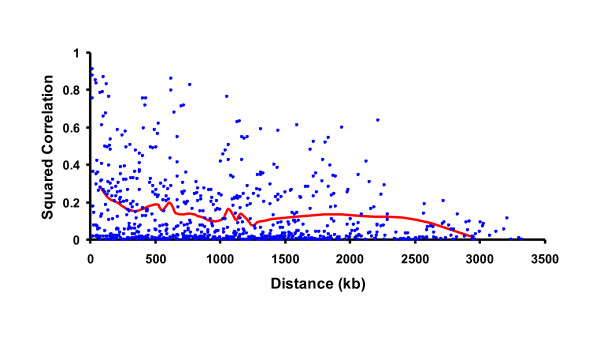
**Linkage pattern from actual Holstein genotypes on chromosome 1**.

Examples of actual and simulated LD patterns are in Figures 2 and 3, respectively. Squared correlations from actual or simulated genotypes were about equal on average for markers separated by 10 to 3000 kb, but actual genotypes had a wider range of values with more very high or low squared correlations that continued across more distant markers. Further testing or a modified algorithm may be needed to obtain a closer match. If true LD is higher than simulated, the reliability of genomic predictions should also be higher, but the advantages of higher density would be less if the lower density markers already have strong LD with the QTL.

### Haplotype imputation

Measures of imputation success from 50,000 markers, 500,000 markers, and the two mixed density datasets are in Table 1. Statistics are provided separately for animals with phenotypes in the reference population, labelled old, and animals without phenotypes, labelled young. In the single-density data sets, percentage of missing genotypes was 1.0% originally but after haplotyping only 0.07% were incorrect, i.e. 0.93% of the missing genotypes were imputed correctly. In the two mixed density data sets, 80 to 86% of the markers were missing originally and 93 to 96% of these missing markers were imputed. The remaining 6.4% and 3.3% of alleles in the two datasets that were not observed and not imputed were set to population allele frequency. If only one allele was imputed, allele frequency was substituted for only the other, unknown allele, and these loci counted as half imputed.

**Table 1:** Measures of imputation success for single- and mixed-density data by age group

Markers used		50 K	Mixed	Mixed	500 K
Number of 500 K genotypes		0	1,406	3,798	33,414
	**Age**^**1**^:				
Missing before imputation (%)	all	1	86	80	1
Missing after imputation (%)	all	0.04	6.4	3.3	0.05
Genotype error rate (%)	young	0.03	1.3	0.9	0.03
	old	0.04	3.4	1.7	0.04
Incorrect genotypes (%)	young	0.06	2.6	1.7	0.06
	old	0.08	7.3	3.4	0.08
Incorrect linkage phase (%)	young	0.3	1.9	1.4	0.1
	old	0.4	5.4	2.5	0.2
Incorrect paternity (%)	young	2.0	4.9	5.0	2.5
	old	4.3	7.6	6.2	4.2
Correlation^2 ^(estimated, true genotypes)	all	0.99	0.84	0.93	0.99
Reliability of linear breeding values (%)	young	82.6	83.4	83.7	84.1
Reliability of nonlinear breeding values (%)	young	84.4	85.3	85.6	86.0
Reliability gain (nonlinear), 500 K - 50 K (%)	young	0.0	0.9	1.2	1.6

^1^old are animals with phenotypes or progeny; young are animals without

Many non-genotyped ancestors with 100% of markers missing originally had sufficiently accurate imputed data to meet the 90% call rate required for genotyped animals. Thus, 1,117 ancestors could have their imputed genotypes included in the genomic evaluation. Nearly all of those animals were dams because most sires were already genotyped. Imputation of the remaining non-genotyped sires was difficult because they had few progeny and because most dams of their progeny were not genotyped.

Paternal alleles were determined incorrectly for about 2% of the heterozygous markers for young animals and for about 4% for old animals in the single-density data. Rates of incorrect paternal allele calls were low because nearly all sires were genotyped, but increased to about 5% for young and 7% for old animals in the mixed-density data. The most popular sires and dams had 100% correctly called linkage phases and paternal alleles, whereas animals with fewer close relatives had somewhat fewer correct calls. Linkage phase was determined incorrectly for less than 2% of the adjacent pairs of heterozygous markers, except for old animals in the mixed-density data when only young animals had been genotyped at higher density. Five percent or fewer of the missing high-density marker genotypes were imputed incorrectly.

The most frequent individual haplotype within a segment was observed on average 5,883 times and accounted for 8.8% of all haplotypes in the population. The most frequent estimated haplotypes were also the most frequent true haplotypes, and their frequencies were similar, averaging 9.2% true vs. 8.8% estimated frequency of the most common haplotype. High frequencies for fairly long haplotypes are not surprising given the pedigree structure and large contributions from popular sires in the recent past.

Numbers of estimated haplotypes averaged 6,627 per 500-marker segment and were very consistent across segments with a SD of only 229. Numbers of true haplotypes averaged 2,735 and were smaller than estimated, possibly because genotyping errors inflated the estimated counts. Numbers of estimated haplotypes decreased to an average of 5,092 per 100-marker segment used with the 50 K single-density data, but the SD increased to 318. The number of potential haplotypes was 66,828 with two haplotypes per animal and 33,414 animals, as compared to only 6,627 observed. Thus, each estimated haplotype was observed about 10 times on average.

With real genotypes, large numbers of haplotypes in a particular segment can indicate regions that are more heterozygous, regions with higher recombination rate such as the pseudo-autosomal region of the X chromosome [27], misplaced markers on the chromosome map, or genotyping errors. Any markers placed by mistake on the wrong chromosome would generate high crossover rates with "adjacent" markers and seriously reduce the efficiency of haplotyping.

### Computation required

Time and memory requirements using one processor were reasonable for all steps with 500,000 markers and are summarized in Table 2. Computations were performed on an Intel Nehalem-EX 2.27 Ghz processor. Simulation of the genotypes required 1.8 hours and 39 gigabytes memory. Storage of the resulting genotypes required 13 gigabytes for 500,000 markers; however, storage of haplotypes required only 2.5 gigabytes. The shared haplotypes were stored just once, and only index numbers were stored for individuals instead of full haplotypes. For the mixed density datasets, only the observed genotypes and the imputed haplotype index numbers were stored, rather than the imputed genotypes, which greatly decreased storage requirements.

**Table 2:** Storage, memory, and time required for each step using one processor

Processing step	Gbytes	CPU hours
Simulation of genotypes	39	1.8
Population haplotyping	2	1.2
Pedigree haplotyping	3	1.8
Iteration for allele effects	8	30
Storage of genotypes	13	-
Storage of haplotypes	3	-

Haplotyping required two hours and 0.6 gigabytes of memory with 50,000 markers and 100 markers per segment for 33,414 animals. Time increased only to 2.5 hours and 3 gigabytes memory with 500,000 simulated markers and 500 markers per segment for this same population. Computing time increased much less than linearly with number of markers because most haplotypes were excluded as not matching after checking just the first few markers in the segment. Time was about equally divided between population and pedigree haplotyping steps, and memory required was about the same for each.

Genomic evaluation required 8 gigabytes of memory and 30 hours to complete 150 iterations for five replicates with 500,000 markers. Convergence was poor for the highly correlated marker effects but was acceptable for the breeding value estimates. Squared correlations of true and estimated breeding values increased by < 0.1% after 150 iterations on average across replicates. Variance of the change in GEBV from consecutive iterations was about .00004 of the variance of GEBV at 150 iterations.

### Genomic reliability

Reliability of GEBV from the nonlinear model averaged 86.0% for young bulls when all animals were genotyped with 500,000 markers as compared with 84.4% using a 50,000-marker subset. This 1.6% reliability increase is similar to that obtained by doubling the number of markers from 20,000 to 40,000 with real data [3] and indicates diminishing returns from greater marker density. The computed reliability from 8,974 bulls plus 4,348 cows and 50,000 simulated markers is 18.1% higher than the 66.3% obtained from 2,175 bulls in an earlier simulation using similar methods [19], and is consistent with continued strong gains from more actual reference animals in both North America and Europe [12].

Table 1 shows results from the analysis of the two mixed densities as well as those from 50,000 or 500,000 single density datasets using the same five data replicates. Genotyping 1,406 bulls at higher density gave about half of the increase in reliability as genotyping all of the 33,414 animals at higher density. Initially, 86% of genotypes were missing, but only 6% of genotypes were missing after haplotyping. With 3,726 bulls, reliability increased to 85.6% and the gain was 75% of that from genotyping all animals at high density.

Reliabilities from a linear model with normal prior were about 1.5% lower than those from the nonlinear model with a heavy-tailed prior for both the 50 K and 500 K simulated data. Optimum parameter values for the prior distribution were about 2 with 50 K data and 4 with 500 K data, much higher than the 1.12 reported by Cole et al. [28] from actual 50 K data. In linear models, the parameter equals 1.0. Advantages from nonlinear models averaged slightly more than those reported by Cole et al. [28] and did not increase with 500 K data, perhaps because adjacent markers are highly correlated within breeds and large numbers of QTL with small effects on traits make isolation of individual marker effects difficult. Harris and Johnson [8] reported no advantage from nonlinear models for higher-density, within-breed simulated data. Larger advantages would be expected if only a few large QTL were simulated, as in Meuwissen and Goddard [9]. If causative mutations become known, chips could be redesigned to genotype these directly instead of increasing density for all regions equally. Until now, patents have excluded known QTL from chip designs.

**Figure 4:**
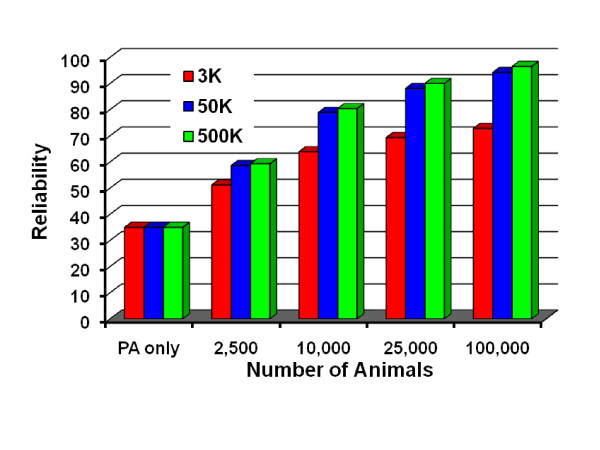
**Expected reliabilities by number of bulls in reference population using 3,000, 50,000, or 500,000 SNP**.

Reliabilities expected with larger reference populations and larger marker densities are in Figure 4. Expectations in the graph are for yield traits using a single density, but combined densities instead allow genotypes to be imputed, bringing reliabilities much closer to those possible when all animals are genotyped at highest density. The graph reflects the 1.6% increase in reliability observed in this simulation. A larger reliability increase was expected from the 10% polygenic variance assumed in U.S. 50,000 marker evaluations. Reliability from 3,000 markers is based on previous studies of actual genotypes [29,30].

Calculations to obtain the REL in Figure 4 were as follows. For the 13,322 reference animals (proven bulls and cows), REL_trad _averaged 87%, REL_pa _averaged 35%, the sum of REL_trad _minus REL_pa _was 13322(.87 - .35) = 6927, and the variance ratio assumed was 15. For the GEBV of young animals, the observed REL_tot _was 84.0% with 500,000 markers. Removal of the contribution from PA reduced this slightly to 82.5%. The remaining polygenic variation not captured by the 500,000 markers was not estimated but assumed to be only 1%. Thus, DE_max _equalled 15(.825/.99)/(1 - .825/.99) = 74.8 and from this the value of *n *was 1389.

The REL_tot _expected from different reference populations and marker numbers were calculated as follows. With 50,000 instead of 500,000 markers, DE_max _is the same but REL_max _from the observed reference population after removing the contribution from REL_pa _was 80.5% instead of 82.5%. This difference in REL_max _gave a solution for *poly *of 1 - .99(.805/.825) = 3.4% with 50,000 markers instead of 1% assumed with 500,000 markers. Similar math applied to REL_max _from 3,000 vs. 43,000 markers with real data in another study [29] gave a solution for *poly *of 30%. Those values of *poly *produced the differing REL_tot _expected with 3,000, 50,000, or 500,000 markers, for example 72.8%, 94.3%, 96.5%, respectively, with 100,000 animals in the reference population. Methods to estimate proportions of correctly called genotypes or squared correlations of estimated and true genotypes are needed for individual animals so that REL_snp _can be included in the published REL.

## Discussion

### Genomic reliability

Observed reliabilities from actual genotypes may be lower than those from simulation [3] and are affected by the distribution of QTL effects, LD among markers, and selection within the population. Current results differ slightly from those reported earlier by VanRaden [31] because of improvements to the haplotyping algorithm, changes to the initial LD and crossover rate simulated, and optimization of the prior parameter for the nonlinear model. With linear mixed models, computation could be greatly reduced using eigenvectors and eigenvalues [32] so that marker equations within chromosomes are diagonal [33]. Reliability gains from increasing marker density in the single breed simulated were small but could be larger if marker effects were estimated from multiple-breed data. The LD of QTL with adjacent markers is not well preserved across breeds with 50,000 markers but should be with 500,000 markers [34]. Thus, higher density genotypes may be more valuable for across than within-breed selection [21]. Pedigrees are not recorded for many animals in actual populations, and much of this information can be recovered even using low density genotyping.

### Computation

Algorithms for imputation are rapidly evolving to meet the demands of growing genomic datasets. Several programs such as those tested by Weigel et al. [6] are available and may provide similar or better results with fewer markers or animals, but most were not designed for very large populations or very dense markers. Fortran program findhap.f90 requires little time and memory and is available at http://aipl.arsusda.gov/software/index.cfm for download. Official genomic evaluations of USDA have used findhap.f90 to impute and include genotypes of dams since April 2010 and 3,000-marker genotypes since December 2010.

Further improvements to imputation algorithms will increase accuracy and allow smaller fractions of animals to be genotyped at highest density. New methods are needed for combining multiple densities, for example 3,000, 50,000, and 500,000 markers, in the same dataset. During the 5 months of review for this manuscript, version 2 of findhap.f90 was released with better properties than those documented here for version 1. Use of pedigree haplotyping followed by population haplotyping can further improve call rates and reduce error rates with similar computation required (Mehdi Sargolzaei, U. Guelph, personal communication, 2010).

The expense of genotyping 1,000-2,000 animals at higher density can be justified for a large population such as Holstein, but larger benefits may be needed if similar numbers are required within each breed. Experimental design is becoming a more important part of animal breeding to balance the speed, reliability and cost of selection. With many new technologies and options available, breeders and breeding companies need accurate advice on the potential of each investment to yield returns. Costs of genotyping are decreasing rapidly, and imputation using less dense marker sets allows the missing genotypes to be obtained almost for free.

## Conclusions

Genotypes and genomic computations are rapidly expanding the data and tools available to breeders. Very high marker density increases reliability of within-breed selection slightly (1.6%) in simulation, whereas lower densities allow breeders to apply cost-effective genomic selection to many more animals. Numbers of reference animals affect reliability more than number of markers, and animals with imputed genotypes contribute to the reference population. New methods for combining information from multiple data sets can improve gains with less cost. Individual reliabilities can be adjusted to account for the number of markers and the accuracy of imputation. More precise estimates of reliability allow breeders to properly balance benefits vs. costs of using different marker sets.

Computer programs that combined population haplotyping with pedigree haplotyping performed well with mixtures of 500,000 and 50,000 marker genotypes simulated for subsets of 33,414 animals. Population haplotyping methods rapidly matched DNA segments for individuals with or without genotyped ancestors, and pedigree haplotyping efficiently imputed genotypes of the non-genotyped parents and correctly filled most missing alleles for progeny genotyped with lower marker density. Accurate imputation can give breeders more reliable genomic evaluations on more animals without genotyping each for all markers.

## List of abbreviations used

b: intercept (genetic base); BV: true breeding value; DE_max_: genomic daughter equivalents with all markers observed; DE_trad_: traditional daughter equivalents; DE_gen_: reduced daughter equivalents from genomics; **e**: vector of errors; GEBV: genomic estimated breeding value; *k*: ratio of error to sire variance; *n*: equivalent reference size needed to achieve 50% genomic reliability; **p**: vector of polygenic effects for each genotyped animal; *poly*: ratio of polygenic variance to additive genetic variance; REL_max_: maximum genomic reliability for an animal with all markers observed; REL_pa_: reliability of parent average; REL_prog_: reliability from own records and progeny; REL_snp_: squared correlation between estimated and true genotypes averaged across loci for each animal; REL_tot_: animal's total reliability from all sources; REL_trad_: reliability of traditional evaluation; **u**: vector of allele effects; X: incidence matrix (= 1) for intercept; **y**: vector of observations; Z: matrix of genotypes minus twice the base allele frequency; σ_a_^2^: additive genetic variance.

## Competing interests

The authors declare that they have no competing interests.

## Authors' contributions

PV derived and programmed the algorithms and drafted the paper. JO and GW suggested several improvements to the imputation methods. KW reviewed available imputation algorithms and suggested experimental designs. All authors read and approved the final manuscript.
